# Deep Learning Analysis of Echocardiographic Images to Predict Positive Genotype in Patients With Hypertrophic Cardiomyopathy

**DOI:** 10.3389/fcvm.2021.669860

**Published:** 2021-08-27

**Authors:** Sae X. Morita, Kenya Kusunose, Akihiro Haga, Masataka Sata, Kohei Hasegawa, Yoshihiko Raita, Muredach P. Reilly, Michael A. Fifer, Mathew S. Maurer, Yuichi J. Shimada

**Affiliations:** ^1^Division of Cardiology, Department of Medicine, Columbia University Irving Medical Center, New York, NY, United States; ^2^Department of Cardiovascular Medicine, Tokushima University, Tokushima, Japan; ^3^Department of Medical Image Informatics, Graduate School of Biomedical Sciences, Tokushima University, Tokushima, Japan; ^4^Department of Emergency Medicine, Massachusetts General Hospital, Boston, MA, United States; ^5^Irving Institute for Clinical and Translational Research, Columbia University Irving Medical Center, New York, NY, United States; ^6^Cardiology Division, Department of Medicine, Massachusetts General Hospital, Boston, MA, United States

**Keywords:** hypertrophic cardiomyopathy, echocardiography, deep learning, genotype, prediction

## Abstract

Genetic testing provides valuable insights into family screening strategies, diagnosis, and prognosis in patients with hypertrophic cardiomyopathy (HCM). On the other hand, genetic testing carries socio-economical and psychological burdens. It is therefore important to identify patients with HCM who are more likely to have positive genotype. However, conventional prediction models based on clinical and echocardiographic parameters offer only modest accuracy and are subject to intra- and inter-observer variability. We therefore hypothesized that deep convolutional neural network (DCNN, a type of deep learning) analysis of echocardiographic images improves the predictive accuracy of positive genotype in patients with HCM. In each case, we obtained parasternal short- and long-axis as well as apical 2-, 3-, 4-, and 5-chamber views. We employed DCNN algorithm to predict positive genotype based on the input echocardiographic images. We performed 5-fold cross-validations. We used 2 reference models—the Mayo HCM Genotype Predictor score (Mayo score) and the Toronto HCM Genotype score (Toronto score). We compared the area under the receiver-operating-characteristic curve (AUC) between a combined model using the reference model plus DCNN-derived probability and the reference model. We calculated the *p*-value by performing 1,000 bootstrapping. We calculated sensitivity, specificity, positive predictive value (PPV), and negative predictive value (NPV). In addition, we examined the net reclassification improvement. We included 99 adults with HCM who underwent genetic testing. Overall, 45 patients (45%) had positive genotype. The new model combining Mayo score and DCNN-derived probability significantly outperformed Mayo score (AUC 0.86 [95% CI 0.79–0.93] vs. 0.72 [0.61–0.82]; *p* < 0.001). Similarly, the new model combining Toronto score and DCNN-derived probability exhibited a higher AUC compared to Toronto score alone (AUC 0.84 [0.76–0.92] vs. 0.75 [0.65–0.85]; *p* = 0.03). An improvement in the sensitivity, specificity, PPV, and NPV was also achieved, along with significant net reclassification improvement. In conclusion, compared to the conventional models, our new model combining the conventional and DCNN-derived models demonstrated superior accuracy to predict positive genotype in patients with HCM.

## Introduction

Hypertrophic cardiomyopathy (HCM) is the most common genetic cardiac disease, affecting ~1 in 200–500 people (1). HCM is caused by mutations in the genes coding for proteins constructing the contractile apparatus of the myocardium (2). Investigators have documented dozens of genes and >1,000 gene mutations associated with HCM pathogenesis (2). Genetic testing has now become a powerful tool for family screening, diagnosis, and prognostication in HCM (3, 4). For example, genetic testing can determine whether each of the first-degree relatives is at risk of developing HCM (3, 5). Genetic testing can also help clinicians establish the diagnosis of HCM in patients with atypical clinical features (5). Furthermore, positive genotype carries a significant prognostic impact (6). On the other hand, genetic testing is time- and resource-intensive, and can introduce substantial financial (7, 8), social (e.g., insurability) (9), and psychological burdens (10). Thus, it is important to precisely determine the pre-test probability in each patient with HCM prior to performing genetic testing.

Several prediction tools have been developed to predict positive genotype in HCM—e.g., the Mayo HCM Genotype Predictor score (“the Mayo score” in this manuscript), the Toronto HCM Genotype score (“the Toronto score”) (11, 12). These scoring systems are based on a limited number of clinical parameters including echocardiographic features [e.g., left ventricular (LV) wall thickness, interventricular septal morphology] (11, 12). However, these measurements can be subjective and are prone to intra- and inter-observer variability. Further, these scoring systems only offer limited predictive accuracy (11–15).

Deep learning is a rapidly evolving approach in a variety of medical settings including cardiovascular imaging (16–20). This technology has the potential to overcome the aforementioned human limitations (21). In the HCM population, a previous study demonstrated that deep learning-derived classification model using echocardiographic images can distinguish HCM from other cardiovascular diseases (22). Nonetheless, no previous studies examined the ability of deep learning to predict positive genotype in HCM. We therefore designed the present study to investigate, in patients with HCM, whether deep convolutional neural network (DCNN, a type of deep learning) analysis of echocardiographic images improves the ability to predict positive genotype compared to the conventional models based only on clinical parameters.

## Methods

### Study Design and Population

We prospectively enrolled patients who were seen at the Center for Advanced Cardiac Care at Columbia University Medical Center (New York, NY, USA) and ≥18 years of age with a clinical diagnosis of HCM between 1988 and 2018. We diagnosed HCM if there was echocardiographic evidence of LV hypertrophy—i.e., max LV wall thickness ≥15 mm—out of proportion to systemic loading conditions and a non-dilated LV (3, 23, 24). We excluded patients based on the following criteria; (1) Patients who have never had genetic testing; (2) Patients with HCM phenocopies such as Fabry disease and cardiac amyloidosis confirmed with appropriate testing (3); and (3) Patients who underwent septal reduction therapy—i.e., septal myectomy, alcohol septal ablation—or heart transplant before enrollment. We collected baseline characteristics of the study sample including medical and family history, medication use, and echocardiographic parameters at the time of genetic testing. The institutional review boards of Columbia University Irving Medical Center and Tokushima University Hospital approved this study.

### Outcome Measure

The primary outcome was positive genotype. By convention, variants categorized as “definitely pathogenic” or “likely pathogenic” were regarded positive in the present analysis (6, 11, 12). Variants classified as “variant of uncertain significance,” “likely benign,” or “benign” were considered negative (6, 11, 12). This definition of positive genotype was used in the present study because only these mutations are clinically actionable (i.e., allow treating physicians and the proband's family members to proceed with cascade genetic screening) and carry diagnostic and prognostic impact (3–5). All the patients were offered genetic testing for HCM using one of the commercially available testing kits (e.g., GeneDx, Invitae). Genetic testing kit was chosen based on available insurance reimbursement and patient preference. A sensitivity analysis was also performed after excluding patients with variant of uncertain significance.

### The Reference Models

We used 2 reference models: (1) the Mayo score and (2) the Toronto score. To calculate the Mayo score, we assigned 1 point for the presence of the following variables: age at diagnosis ≤45 years, maximal LV wall thickness ≥20 mm on transthoracic echocardiography, reverse curve septal morphology on transthoracic echocardiography, positive family history of HCM, and positive family history of sudden cardiac death (11). We subtracted 1 point from the score if hypertension was present (11). We made the diagnosis of hypertension based on past medical history, blood pressure measurements, and antihypertensive medication use. We did not count antihypertensives solely used for non-antihypertensive purposes—e.g., β-blockers and/or non-dihydropyridine calcium channel blockers for symptomatic relief of obstructive HCM and/or rate control of atrial fibrillation. For the calculation of the Toronto score, we used the following weighted variables: age at diagnosis, sex, hypertension, family history of HCM, septal morphological subtype (i.e., reverse or neutral), and the ratio of maximal LV wall thickness to posterior wall thickness (12).

### Acquisition of Echocardiographic Images

Standard echocardiographic examinations were performed using a commercially available ultrasound system (iE33, Philips Healthcare, Amsterdam, The Netherlands) as a part of routine clinical care according to the guideline recommendations (25). The 2-dimensional echocardiographic images of all subjects were obtained from the parasternal short- (SAX) and long-axis (LAX) views as well as the apical 2- (AP2), 3- (AP3), 4- (AP4), and 5-chamber (AP5) views. We selected cases with good or adequate imaging quality on the basis of the visualization of the LV walls and endocardial borders. Echocardiographic images were stored digitally as a DICOM file and analyzed offline.

### Import of the Echocardiographic Images

Echocardiographic images from the SAX, LAX, AP2, AP3, AP4, and AP5 views were analyzed. All DICOM images were rigidly registered and rescaled into a reference image to adjust the size of the echocardiographic images. The images were cut and down-sampled to 18.07 × 18.07 cm with 120 × 120 monochrome pixels. Simultaneously, metadata presented in the periphery of the images were removed. To adjust for differences in frame rate and heart rate between patients, 10 equally-spaced images per 1 cardiac cycle were chosen with the use of a semi-automatic heartbeat analysis algorithm. The starting frame was defined by the R wave on the electrocardiogram as a recording of echocardiographic images are triggered by the R wave. The methodological details are provided in Supplementary Methods and have been published previously (16).

### Deep Learning Algorithm

**Figure 1:**
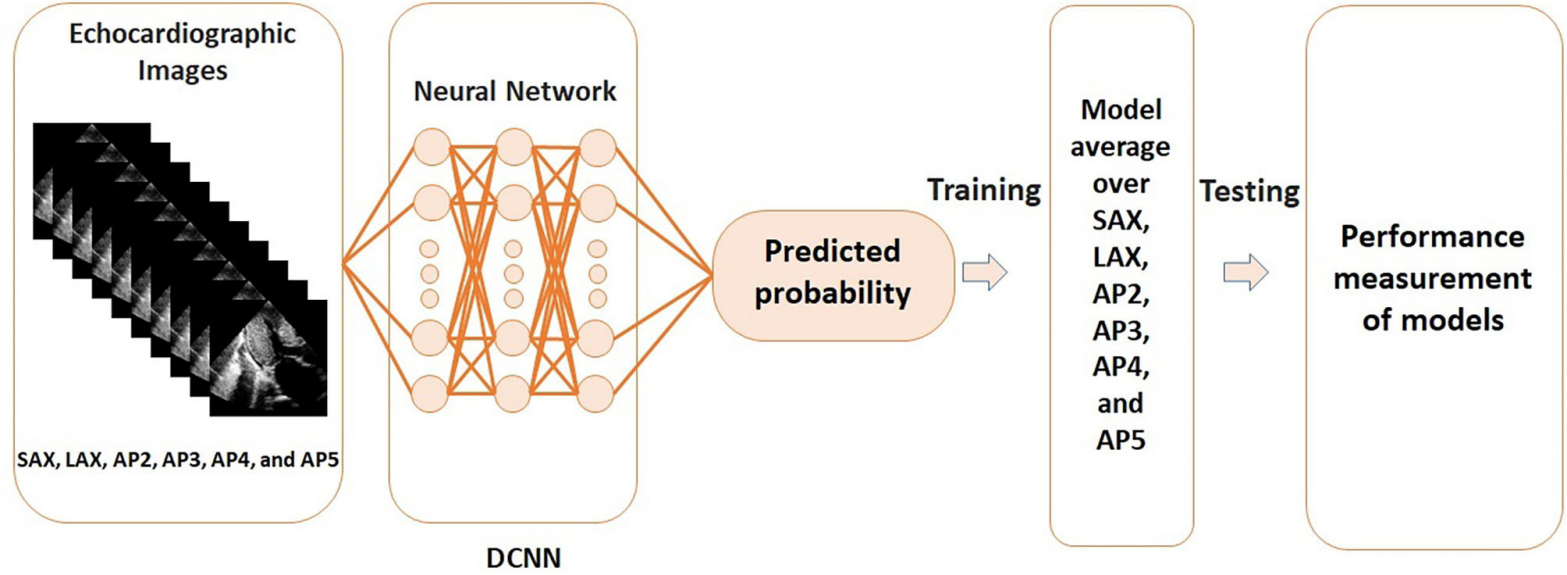
Steps of the deep convoluted neural network analysis using echocardiographic images. DCNN, deep convoluted neural network; AP2, apical 2-chamber view; AP3, apical 3-chamber view; AP4, apical 4-chamber view; AP5, apical 5-chamber view; LAX, parasternal long-axis view; SAX, parasternal short-axis view.

Figure 1 visualizes the processing steps of DCNN. Positive genotype was predicted by a DCNN algorithm using the 6 views (SAX, LAX, AP2, AP3, AP4, and AP5). All data were randomly divided into 5 groups and 4 of the groups were used as the training set to develop the model, and the rest was used as the test set to examine the model performance (i.e., 5-fold cross-validation; Supplementary Figure 1). To avoid an unexpected extraction of undesired features for the evaluation, training data were augmented in each dataset. The output was the probability of positive genotype. Model training was performed on a graphics processing unit (GeForce GTX 1080 Ti, NVIDIA, Santa Clara, California, USA). The Adam optimizer was used for training (Supplementary Figure 2) (26). The details are provided in Supplementary Methods. Deep learning was performed with the Python 3.6 programming language with Keras 2.1.5. Additionally, to visually display which part of the heart the DCNN-based models were focused on, gradient-weighted class activation mapping (grad-CAM) analysis was performed (27).

### Statistical Analysis

For comparisons of the baseline characteristics between patients with positive and negative genotype, Fisher's exact test, Student's *t*-test, or chi-squared test was used, as appropriate. The following steps were taken to compare the area under the receiver operating characteristics curve (AUC) of one of the reference models (i.e., the Mayo score or the Toronto score) and that of a new model combining the reference model with the DCNN-derived model. First, logistic regression model was constructed to estimate the coefficient values and the constant to combine the reference model and the DCNN-derived probability. Second, the AUC of the reference model and that of the combined model were compared using non-parametric receiver operating characteristic estimation with 1,000 bootstrapping. The Stata command *rocreg* with *auc* option was used to perform this step. Additionally, the net reclassification improvement was examined using the Stata command *incrisk*. The sensitivity, specificity, positive predictive value (PPV), and negative predictive value (NPV) were also calculated. Statistical significance was declared if the 2-sided *p*-value was <0.05. These analyses were performed using Stata Statistical Software: Release 12 (StataCorp LP, College Station, TX).

## Results

Initially, 105 patients with HCM who underwent genetic testing and had at least 1 echocardiographic study were screened. In this cohort, six patients were excluded based on the exclusion criteria. The most common reasons for exclusion were prior septal reduction therapy and prior heart transplant. As a result, 99 patients were included in the analysis. A total of 45 (45%) patients had positive genotype. This proportion is similar to what has been reported in the literature (6). Baseline patient characteristics are shown in Table 1. Patients with positive genotype were younger and more likely to have family history of HCM as well as reverse septal contour, and had lower systolic blood pressure.

**Table 1:** Baseline clinical characteristics of the study sample.

Characteristics^*^	**Genotype**		
	**Positive**	**Negative**	***P*-value**
	***n* = 45**	***n* = 54**	
**Demographics**			
Age (year)	40 ± 17	55 ± 22	<0.001
Female	17 (38)	18 (33)	0.80
NYHA functional class	1 [1–2]	1 [1–2]	0.70
Race/ethnicity			0.77
Caucasian	36 (80)	46 (85)	
African-American	1 (2)	2 (4)	
Asian	1 (2)	0 (0)	
Other or unidentified	7 (16)	6 (11)	
**Medical history**			
Prior AF	26 (58)	29 (54)	0.83
Prior sustained VT/VF	8 (18)	3 (6)	0.10
Prior non-sustained VT	13 (29)	9 (17)	0.49
Prior syncope	9 (20)	6 (11)	0.34
Family history of sudden cardiac death	10 (22)	7 (16)	0.34
Family history of HCM	21 (47)	6 (13)	<0.001
**Medications**			
β-blocker	26 (58)	29 (54)	0.83
Calcium channel blocker	11 (24)	21 (39)	0.19
ACE inhibitor	2 (4)	8 (15)	0.10
ARB	1 (2)	10 (19)	0.02
Diuretic			
Loop diuretic	4 (9)	7 (13)	0.74
Thiazide	1 (2)	11 (20)	0.01
Potassium-sparing diuretic	1 (2)	7 (13)	0.07
Disopyramide	17 (38)	5 (9)	0.001
Amiodarone	2 (4)	4 (2)	0.69
**Echocardiographic measurements**			
Left atrial diameter (mm)	43 ± 8	44 ± 8	0.65
Systolic blood pressure (mmHg)	117 ± 17	129 ± 19	0.001
Diastolic blood pressure (mmHg)	71 ± 10	74 ± 11	0.17
Interventricular septum thickness (mm)	19 ± 5	18 ± 5	0.38
Posterior wall thickness (mm)	12 ± 3	13 ± 3	0.08
Left ventricular outflow tract gradient (mmHg) at rest	18 [0–25]	26 [0–40]	0.18
Left ventricular outflow tract gradient (mmHg) with Valsalva maneuver	35 [0–51]	38 [0–64]	0.79
Left ventricular ejection fraction (%)	60 ± 10	60 ± 12	0.97
Left ventricular end-diastolic diameter (mm)	43 ± 7	44 ± 9	0.65
Left ventricular end-systolic diameter (mm)	29 ± 7	28 ± 9	0.80
Systolic anterior motion of mitral valve leaflet	24 (53)	29 (54)	>0.99
Degree of mitral regurgitation^†^	1 [1–2]	1 [1–2]	0.67
**Genotype**			–
MYBPC3	20 (44)	–	
MYH7	12 (27)	–	
TNNT2	5 (11)	–	
MYL2	3 (7)	–	
ACTN2	1 (2)	–	
THBD	1 (2)	–	
Multiple	1 (2)	–	
Other	2 (4)	–	
**Predictors of positive genotype**			
Age at diagnosis	34 ± 17	49 ± 21	<0.001
Reverse septal contour	21 (47)	13 (24)	0.03
Maximal left ventricular wall thickness	19 ± 5	18 ± 5	0.38
Hypertension	26 (58)	34 (63)	0.74
Ratio of maximal wall thickness to posterior wall thickness			0.03
<1.46	16 (36)	33 (61)	
1.47–1.70	13 (29)	9 (17)	
1.71–1.92	9 (20)	5 (9)	
1.93–2.26	4 (9)	4 (7)	
>2.27	3 (7)	3 (6)	

* *Data were expressed as number (percentage), mean ± standard deviation, or median [interquartile range]*.

† *Degree of mitral regurgitation was converted to numerical values according to the following rule: none = 0, trace = 1, trace to mild = 1.5, mild = 2, mild to moderate = 2.5, moderate = 3, moderate to severe = 3.5, severe = 4*.

*ACE, angiotensin converting enzyme; AF, atrial fibrillation; ARB, angiotensin II receptor blocker; HCM, hypertrophic cardiomyopathy; NYHA, New York Heart Association; VT/VF, ventricular tachycardia or ventricular fibrillation*.

**Figure 2:**
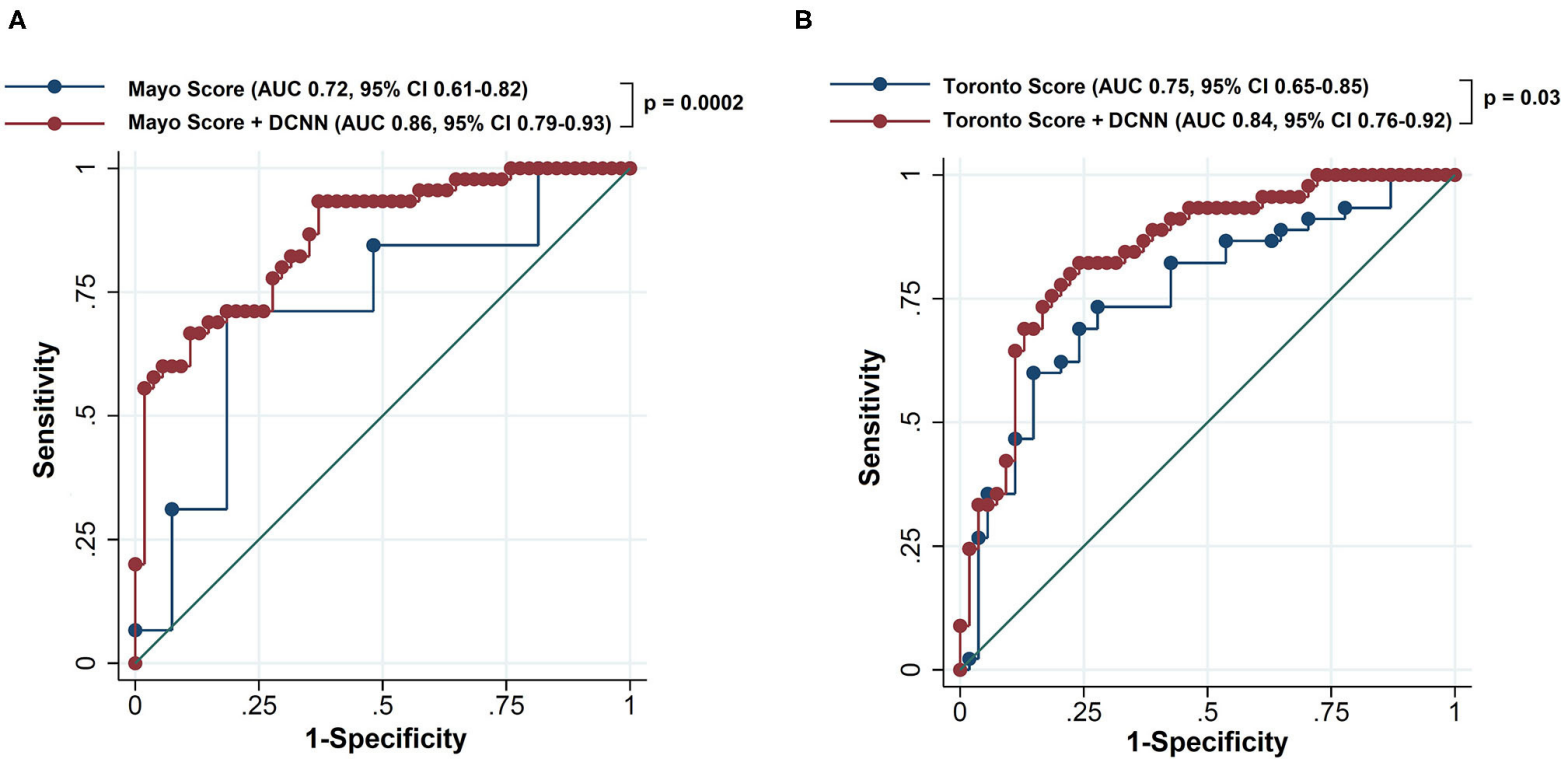
Receiver operating characteristics curve of the reference scoring system and new model combining the reference scoring system and deep convolutional neural network-based probability to predict positive genotype in patients with hypertrophic cardiomyopathy. The reference scoring system was the Mayo score in **(A)** and the Toronto score in **(B)**. The dots represent different threshold levels. DCNN, deep convoluted neural network; AUC, area under the receiver operating characteristic curve; CI, confidence interval.

The DCNN-predicted probability showed the AUC of 0.76 (95% CI 0.66–0.86). The AUC of the Mayo score was 0.72 (95% CI 0.61–0.82). Table 2 summarizes the net reclassification improvement, sensitivity, specificity, PPV, and NPV using the Mayo score as the reference model. The new model combining the Mayo score with the DCNN-predicted probability significantly improved the predictive accuracy compared to the Mayo score (AUC = 0.86; 95% CI 0.79–0.93; *p* < 0.001; Figure 2). There was also a significant net reclassification improvement (Table 2), indicating that a larger number of patients were reclassified in the right direction compared to the number of patients who were reclassified in the wrong direction. The coefficients and constant to construct the combined model are shown in Supplementary Results. The sensitivity analysis after excluding patients with variant of uncertain significance showed similar findings; the AUC of the Mayo score was 0.73, whereas that of the combined model was 0.87 (*p* = 0.0002).

**Table 2:** Predictive performance of Mayo score and a new model combining Mayo score and deep convolutional neural network-based probability to predict positive genotype in patients with hypertrophic cardiomyopathy.

**Prediction model**	**AUC**	*P*-value^*^	**NRI^†^**	***P*-value^†^**	**Sensitivity (%)**	**Specificity (%)**	**PPV (%)**	**NPV (%)**
Mayo score (reference)	0.72 (0.61–0.82)	Reference	Reference	Reference	71 (56–84)	81 (69–91)	76 (61–87)	77 (63–88)
Mayo score + DCNN	0.86 (0.79–0.93)	<0.001	0.71 (0.30–1.24)	<0.001	71 (56–84)	81 (69–91)	76 (61–87)	77 (63–88)

* *P-value was calculated to compare AUC of the reference model with that of the combined model*.

† *Continuous NRI and associated p-values were displayed*.

*AUC, area under the receiver-operating-characteristic curve; DCNN, deep convoluted neural network; NPV, negative predictive value; NRI, net reclassification improvement; PPV, positive predictive value*.

When the Toronto score was used as the reference model, the AUC was 0.75 (95% CI 0.65–0.85; Table 3). The new model combining the Toronto score with the DCNN-predicted probability exhibited significant improvement in the AUC compared to the Toronto score alone (AUC 0.84, 95% CI 0.76–0.92, *p* = 0.03; Figure 2). A significant net reclassification improvement was also achieved along with improvement in the sensitivity, specificity, PPV, and NPV (Table 3). After excluding patients with variant of uncertain significance, the AUC of the Toronto score was 0.74 and that of the combined model was 0.85 (*p* = 0.01).

**Table 3:** Predictive performance of Toronto score and a new model combining Toronto score and deep convolutional neural network-based probability to predict positive genotype in patients with hypertrophic cardiomyopathy.

**Prediction model**	**AUC**	*P*-value^*^	**NRI^†^**	***P*-value^†^**	**Sensitivity (%)**	**Specificity (%)**	**PPV (%)**	**NPV (%)**
Toronto score (reference)	0.75 (0.65–0.85)	Reference	Reference	Reference	73 (58–85)	72 (58–84)	69 (54–82)	76 (62–86)
Toronto score + DCNN	0.84 (0.76–0.92)	0.03	0.64 (0.34–1.22)	<0.001	80 (65–90)	78 (64–88)	75 (61–88)	82 (69–91)

* *P-value was calculated to compare AUC of the reference model with that of the combined model*.

† *Continuous NRI and associated p-values were displayed*.

*AUC, area under the receiver-operating-characteristic curve; DCNN, deep convoluted neural network; NPV, negative predictive values; NRI, net reclassification improvement; PPV, positive predictive value*.

**Figure 3:**
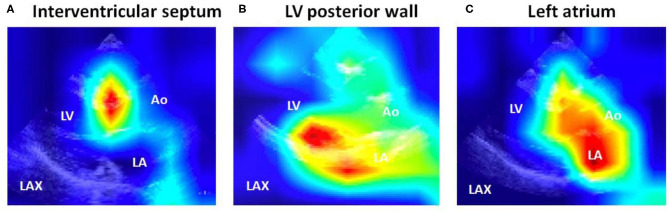
Spatial display of important features detected by the deep convoluted neural network analysis models. Note that the important features were localized to **(A)** the interventricular septum, **(B)** the LV posterior wall, and **(C)** the left atrium. Ao, aorta; LA, left atrium; LAX: parasternal long-axis view; LV, left ventricular/ventricle.

To improve the interpretability of DCNN models, representative visualizations generated by grad-CAM are shown in Figure 3. This visualization method revealed that the DCNN-based models applied a large weight on the LV walls (e.g., the interventricular septum and posterior wall) and the left atrium.

## Discussion

### Summary of Findings

In this study that examined the incremental value of deep learning-based models to predict positive genotype, the predictive ability of our novel models combining the conventional model and the deep learning-based probability significantly outperformed that of the conventional models. The present study serves as the first investigation demonstrating the additional value of deep learning-based analysis of echocardiographic images in predicting positive genotype in patients with HCM.

### Impact of Positive Genotype on Family Screening, Diagnosis, and Prognostication

Genetic testing is useful in determining family screening strategies in HCM. Without genetic testing, first-degree relatives have to undergo phenotypic screening with electrocardiogram and echocardiography every 5 years, and more frequently if the age is <18 years (3, 5). This burden can be relieved if the proband has positive genotype and the family member does not carry the identified gene mutation (3, 5). Furthermore, genetic testing has both diagnostic and prognostic values. In patients with suspected HCM, positive genotype confirms the diagnosis of HCM (2). With regard to prognostication, patients with positive genotype had a 2-fold higher risk of adverse outcomes (e.g., heart failure, atrial fibrillation) compared to those with negative phenotype in a prospective cohort study of patients with HCM (6). Thus, positive genotype can have a substantial impact on the clinical management of patients with HCM and their family members.

On the other hand, genetic testing can carry substantial financial and social burdens. For example, genetic testing costs a few thousand dollars in the US, and the proportion of the patient's out-of-pocket payment depends on the insurance type and plan. With regard to the social burden of genetic testing, while the Genetic Information Non-discrimination Act prohibits discrimination of insurability based on genetic testing results, the law is silent regarding life, disability, and long-term care insurance (9, 28, 29). As such, genetic testing can result in non-negligible burdens, and accurate identification of patients with HCM who have high pre-test probability carries clinical, socio-economical, and psychological importance.

Nevertheless, the currently available conventional models—i.e., the Mayo score, the Toronto score—offer only modest ability to predict positive genotype. The AUCs of these scoring systems have been reported to be ~0.75 (11–15), which is in agreement with those in the present study (0.72 with the Mayo score and 0.75 with the Toronto score). In this context, findings in the current analysis add to the body of knowledge by demonstrating that the deep learning-based analysis of echocardiographic images provides incremental value to the conventional models in predicting positive genotype in patients with HCM.

### Advantages of Deep Learning-Based Approach Over the Conventional Prediction Methods

The Mayo and Toronto scoring systems include a limited number of parameters determined by echocardiography—e.g., maximal LV wall thickness, septal morphological subtypes. However, these parameters have been known to have large intra- and inter-observer variability (11, 30). It is a time- and cost-intensive process to train physicians until they can accurately measure the wall thickness and classify the septal morphology (31). Even after going through such specialized trainings, the interpretation of echocardiographic images still remains interpreter-dependent and subjective, and can be affected by fatigue (31). Moreover, these parameters used in the conventional models do not account for dynamic (i.e., non-static) image information.

By contrast, deep learning has a potential to overcome such variability in human assessment of echocardiographic measurements (32). Deep learning is also able to extract information that is not readily apparent to humans (33). Thus, deep learning-based models can offer a new avenue to generate an accurate, consistent, rapid, and automated interpretation of echocardiographic images while reducing the risk of human errors. Its application has shown a high potential to revolutionize the process of diagnosis and prognostication, with promising results in the fields of dermatology (34), radiology (35), and cardiology (16, 36). In the HCM population, a prior study reported that a deep learning-derived classification model using echocardiographic images can differentiate HCM from cardiac amyloidosis and pulmonary arterial hypertension (22). Furthermore, our DCNN approach utilizes not only spatial but also temporal information by incorporating the additional dimension of time.

Despite the potential usefulness, no prior studies have applied deep learning-based methods to predict positive genotype in HCM. The present analysis represents the first study to exhibit the incremental value of deep learning-based analysis of echocardiographic images in addition to the conventional clinical parameters to predict positive genotype in the HCM population. The ability of our deep learning-based approach to analyze echocardiographic images obtained in routine clinical care—as opposed to “research-quality” images gained for investigational purposes—further underscores the feasibility and generalizability of this novel method.

### Spatial Visualization of Important Features to Identify Genotype-Positive Patients

Deep learning technology is frequently referred to as a black box—i.e., it does not provide information as to which features are mainly used for the development of discrimination models. Our deep learning method is not an exception. To address this issue, in the present study, we have performed the grad-CAM analysis and provided visualization of the important features that the deep learning models focused on, which greatly enhances the interpretability (27). This analysis demonstrated an interesting finding—in addition to the LV, features spatially located in the left atrium were frequently used to distinguish between patients with positive and negative genotype in HCM. This observation is consistent with our prior knowledge; the left atrial diameter has been known to predict sudden cardiac death (37) and cardiovascular death in the HCM population (38). The inferences from our study suggest that echocardiographic parameters related to the left atrium—e.g., left atrial diameter, volume, and ejection fraction—have a potential to predict positive genotype in the HCM population.

### Potential Limitations

Findings in the present study should be interpreted with several limitations in mind. First, the present study is subject to selection bias. The study sample was limited to patients with HCM who underwent genetic testing. Second, positive genotype was defined by the currently available classification of mutations; however, the classification of each mutation can change in the future. Third, validation with external samples was not performed. This study should prompt model validation with a new cohort. Last, the study samples were relatively homogeneous in terms of race and sex. Further, there is a possibility that the spectrum of mutations observed in the study samples may not exactly represent those in the general HCM population. Therefore, generalizability of the results to other HCM populations (e.g., those who are not followed at HCM referral centers) needs to be established.

### Conclusions

Compared to the conventional models based on clinical and echocardiographic parameters, our new models integrating the conventional and deep learning-based analysis of echocardiographic images demonstrated a superior ability to predict positive genotype in patients with HCM. For patients and treating physicians, the novel deep learning-based method introduced in the present study can be used as an assistive technology to inform the decision-making process of performing genetic testing; deep learning coupled to human expertise can provide more accurate pre-test probability. For researchers, the current analysis would prompt further investigation into developing a better deep learning model to predict positive genotype in patients with HCM.

## Data Availability Statement

The original contributions presented in the study are included in the article/Supplementary Materials. Further inquiries can be directed to the corresponding author.

## Ethics Statement

The studies involving human participants were reviewed and approved by the institutional review boards of Columbia University Irving Medical Center and Tokushima University Hospital. The patients/participants provided their written informed consent to participate in this study.

## Author Contributions

SM wrote the manuscript. KK and YS conceived the idea for the manuscript. KK and AH performed the deep learning analyses of echocardiographic images. YS was guarantor and performed the statistical analyses. SM and YS contributed to data acquisition. MS, KH, YR, MR, MF, MM, and YS contributed to interpreting the data and revising the work critically for intellectual content. All authors made the decision to submit.

## Conflict of Interest

The authors declare that the research was conducted in the absence of any commercial or financial relationships that could be construed as a potential conflict of interest.

## Publisher's Note

All claims expressed in this article are solely those of the authors and do not necessarily represent those of their affiliated organizations, or those of the publisher, the editors and the reviewers. Any product that may be evaluated in this article, or claim that may be made by its manufacturer, is not guaranteed or endorsed by the publisher.
