# Supplementary material for: Deep Learning Analysis of Echocardiographic Images to Predict Positive Genotype in Patients With Hypertrophic Cardiomyopathy
Source: Front Cardiovasc Med. 2021 Aug 27;8:669860. doi: 10.3389/fcvm.2021.669860 (PMC8429777; doi:10.3389/fcvm.2021.669860)
Supplement: Supplementary file 1 [file Data_Sheet_1.DOCX]

**SUPPLEMENTAL MATERIAL**

**Deep Learning Analysis of Echocardiographic Images to Predict Positive Genotype
in Patients with Hypertrophic Cardiomyopathy**

Sae X. Morita, MD, MSc, Kenya Kusunose, MD, PhD, Akihiro Haga, PhD,

Masataka Sata, MD, PhD, Kohei Hasegawa, MD, MPH, MS, Yoshihiko Raita MD, MPH, MMSc, Muredach P. Reilly, MB, MS, Michael A. Fifer, MD, Mathew S. Maurer, MD,

and Yuichi J. Shimada, MD, MPH

**Supplemental Methods**

*Data splitting and augmentation*

**Figure S1** illustrates the details of the 5-fold cross-validation and data augmentation. Data augmentation was performed by sliding the initial images. In each patient in the training set (n = 79 or 80), the data were augmented by 10 times for the deep convolutional neural network (DCNN). For example, selected 10 images were regarded as the cyclic ones, and the initial images were re-shifted from T = 0 to T = 9, resulting in augmented data by 10 times. Thus, each training set had approximately 800 datasets.

*Classification model*

**Figure S2** visualizes the DCNN model used in this study, where the network comprised a total of 5 convolutional layers of *N*, 2*N*, 2*N*, 2*N*, and *N* filters with kernel sizes of 3×3×3 and 5 pooling layers of kernel size 2×2×2 were applied. A series of 2 fully connected layers – with 512 nodes and 5 units – were included in the final layer. Leaky Rectified Linear Unit (LeakyRelu) was used as an activation function in all activation layers except for the last activation, where the softmax function was employed. The number of the filters in the layers was controlled by *N*, and *N* = 64 was used in the present study. The models output a probability of positive genotype by 3D images (size of 120×120×10). To adjust for differences in frame rate and heart rate, we selected 10 equally spaced images per 1 cardiac cycle with a semi-automatic heartbeat analysis algorithm.^20^ The model was created for each of 6 views (SAX, LAX, AP2, AP3, AP4, and AP5) independently, and finally the probability was averaged (model average) to predict positive genotype. The cross-entropy error function was used as the loss function to be reduced. The number of iterations (epochs) was set at 50 based on the behavior of the loss function. The weights giving the minimum loss were applied to the test set.

**Supplemental Results**

For the logistic regression model combining the Mayo score with the DCNN-derived score, the coefficient was 0.9724016 for the Mayo score and -7.218164 for the DCNN-derived score, and the constant was 2.486204. For combining the Toronto score with the DCNN-derived score, the coefficient was 0.1574007 for the Toronto score and -7.058067 for the DCNN-derived score, and the constant was 3.233892

**Figure S1. Details of the 5-fold cross-validation and data augmentation**

**
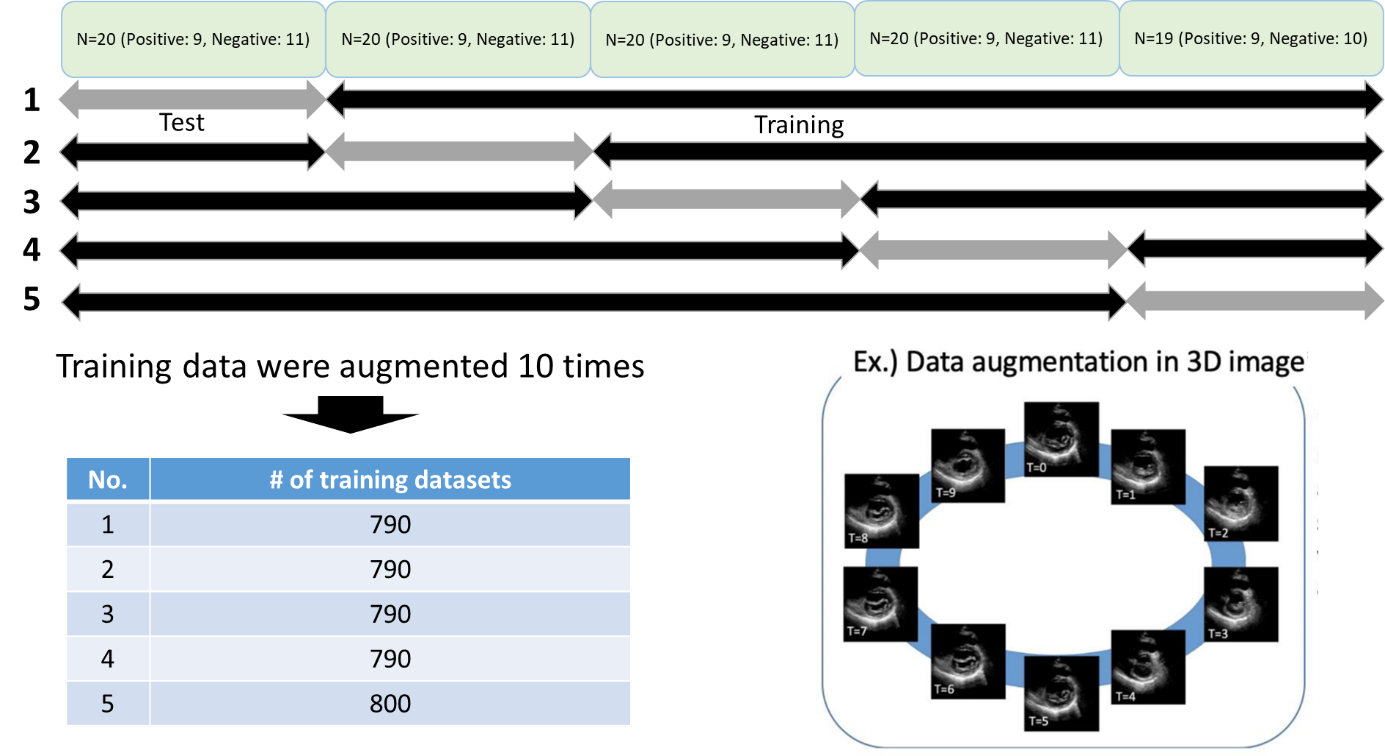
**

**Figure S2. Structure of the deep convolutional neural network models**

**
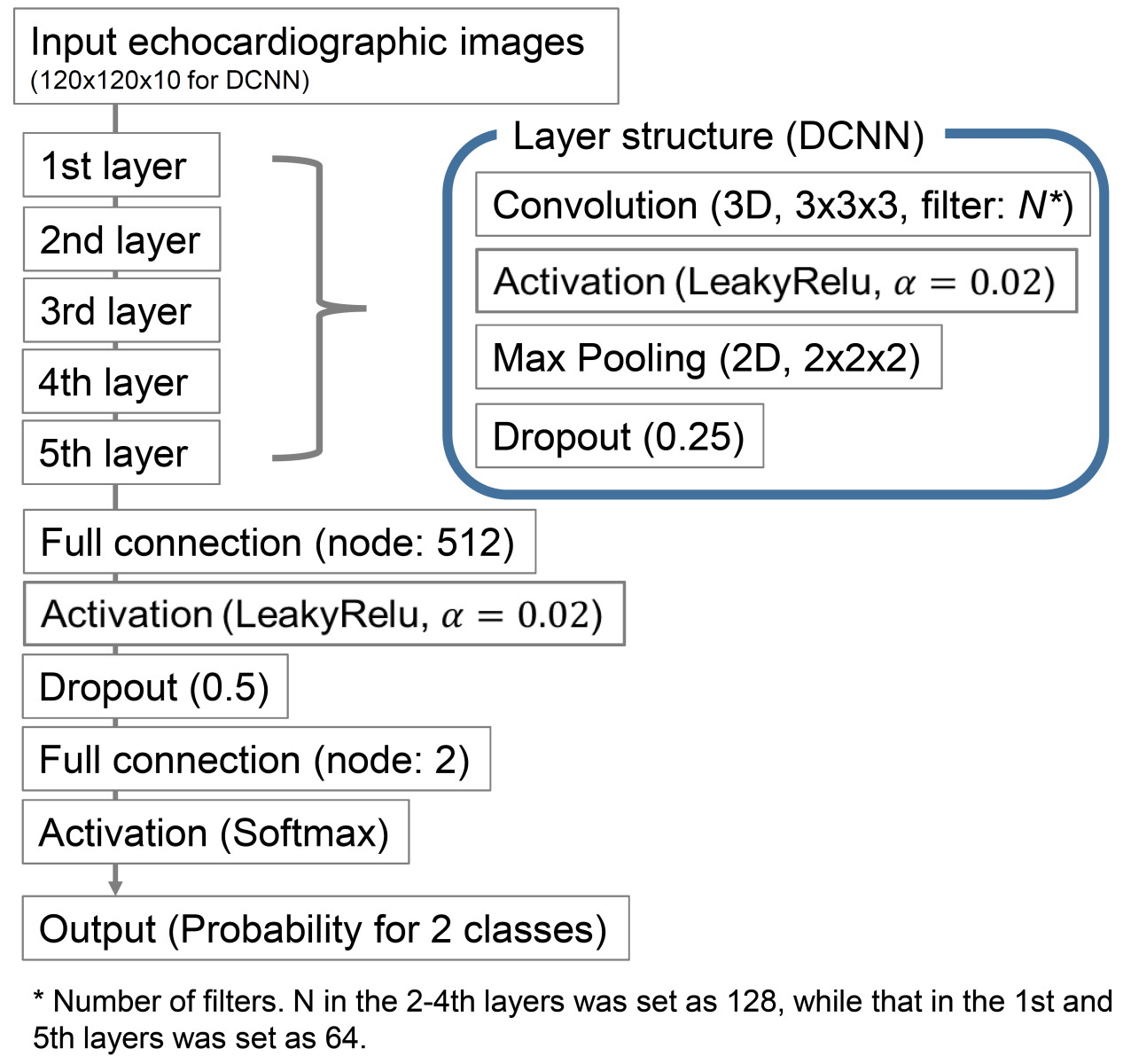
**

Abbreviations: DCNN, deep convoluted neural network
